# Enzymatic Inactivation of Oxysterols in Breast Tumor Cells Constraints Metastasis Formation by Reprogramming the Metastatic Lung Microenvironment

**DOI:** 10.3389/fimmu.2018.02251

**Published:** 2018-10-02

**Authors:** Marta A. Moresco, Laura Raccosta, Gianfranca Corna, Daniela Maggioni, Matias Soncini, Silvio Bicciato, Claudio Doglioni, Vincenzo Russo

**Affiliations:** ^1^Immuno-Biotherapy of Melanoma and Solid Tumors Unit, Division of Experimental Oncology, IRCCS Scientific Institute San Raffaele, Milan, Italy; ^2^Università Vita-Salute San Raffaele, Milan, Italy; ^3^Center for Genome Research, University of Modena and Reggio Emilia, Reggio Emilia, Italy; ^4^Department of Pathology, IRCCS Scientific Institute San Raffaele, Milan, Italy

**Keywords:** oxysterols, sulfotransferases, immune evasion, tumor microenvironment, metastasis

## Abstract

Recent evidence indicates that immune cells contribute to the formation of tumor metastases by regulating the pre-metastatic niche. Whether tumor-derived factors involved in primary tumor formation play a role in metastasis formation is poorly characterized. Oxysterols act as endogenous regulators of lipid metabolism through the interaction with the nuclear Liver X Receptors-(LXR)α and LXRβ. In the context of tumor development, they establish a pro-tumor environment by dampening antitumor immune responses, and by recruiting pro-angiogenic and immunosuppressive neutrophils. However, the ability of LXR/oxysterol axis to promote tumor invasion and metastasis by exploiting immune cells, is still up to debate. In this study we provide evidence that oxysterols participate in the primary growth of orthotopically implanted 4T1 breast tumors by establishing a tumor-promoting microenvironment. Furthermore, we show that oxysterols are involved in the metastatic spread of 4T1 breast tumors, since their enzymatic inactivation mediated by the sulfotransferase 2B1b, reduces the number of metastatic cells in the lungs of tumor-bearing mice. Finally, we provide evidence that oxysterols support the metastatic cascade by modifying the lung metastatic niche, particularly allowing the recruitment of tumor-promoting neutrophils. These results identify a possible new metastatic pathway to target in order to prevent metastasis formation in breast cancer patients.

## Introduction

The role exerted by inflammation in promoting tumor cell formation (neoplastic transformation) as well as the mechanisms of immune escape favoring tumor growth have recently been considered as pillars of cancer hallmarks (1). Emphasis has recently been given to metabolic pathways altered in cancer cells, which may condition tumor-infiltrating immune cells. On the other hand, defined metabolic pathways specific for immune cell subsets have been identified. Metabolites produced by cancer cells may hamper the antitumor immune response by affecting distinct tumor-infiltrating immune cells (2). Among these factors cholesterol metabolites, namely oxysterols, help tumor progression by promoting immunosuppressive networks or angiogenesis. Oxysterols are formed through two different pathways, enzymatic reactions or non-enzymatic reactions initiated by non-radical ROS or by inorganic free-radical species (3, 4). Some oxysterols are generated exclusively by either pathways, while others can be produced by both (5, 6). Oxysterols were identified as ligands of the nuclear Liver X Receptors (LXRs) (7).

LXRα and LXRβ, respectively encoded by NR1H3 and NR1H2 gene, belong to a superfamily of nuclear receptors activated upon ligand binding (8). LXRs function as heterodimers with retinoid X receptors (RXR) and bind to a specific DNA sequence, called LXRE. The activation of LXRs increases the rate of cholesterol efflux from cells via ABCG1 and ABCA1 cholesterol transporters, and lipogenesis mainly through the induction of the sterol regulatory element-binding protein 1c (SREBP-1c) (8). Besides lipid homeostasis, these receptors control carbohydrate and energy metabolism (9). LXRs/LXR ligands/oxysterols integrate cholesterol metabolism and inflammation (10). Indeed, many inflammatory processes and immune cells respond to the modification of this axis through the activation of specific anti-inflammatory programs (11). LXRs/LXR ligands/oxysterols participate in tumor formation directly by promoting primary tumor growth (12) or indirectly by modulating the antitumor immune response (13). In mouse models of melanoma and breast tumors, the activation of LXR signaling in tumor cells and/or in stromal cells was shown to promote invasion and metastasis (14, 15). Specifically, in breast tumors the activation of LXRs was shown to induce epithelial-mesenchymal transition (EMT), ultimately contributing to the formation of lung metastases (15).

Recent evidence indicates that the pre-metastatic sites are shaped before tumor cell arrival to generate a tumor-hospitable niche, which favors the metastatic cell seeding. The pre-metastatic niche is conditioned by hematopoietic-derived cells, such as myeloid cells and/or neutrophils (16, 17). These cells have been detected in the pre-metastatic niche. Wculek and Malanchi have recently reported that neutrophil-derived leukotrienes allow for the colonization of lungs by cancer cells endowed with high tumorigenic potential (17).

Neutrophils are primarily recruited by CXCR2 ligands (e.g., CXCL1, CXCL5, etc.). However, our studies have shown that neutrophils can respond to other chemoattractant stimuli, such as oxysterols (18), which in turn recruit them within the primary tumors and contribute to tumor growth by exerting immune suppressive and/or pro-angiogenic activities (18). Altogether, these results highlight the role played by the cross-talk among cancer cells, stroma and myeloid cells, including neutrophils, in favoring primary and metastatic tumor cell seeding.

Here, we show that tumor-derived oxysterols, interfering with molecular and cellular networks, modify primary and metastatic tumor microenvironment (TME) and participate in the formation of primary and metastatic growth of breast tumors. We detected type-2 inflammation and neutrophil increase in the TME of the 4T1 breast primary tumors. These features were counteracted when we injected mice with 4T1 expressing SULT2B1b, which inactivates oxysterols. In these mice, we detected a shift toward type-1 inflammation concomitant with neutrophil reduction, DC increase and a high CD8^+^IFNγ^+^/CD4^+^IL-4^+^ ratio. In the metastatic lungs, we observed a reduction of neutrophils and monocytes, as compared to lungs from mock-4T1-bearing mice. Of note, a similar difference was observed when we injected mice intraperitoneally with conditioned medium from mock- or SULT2B1b-4T1.

## Results

### Role of oxysterols in regulating primary breast tumor growth

**Figure 1:**
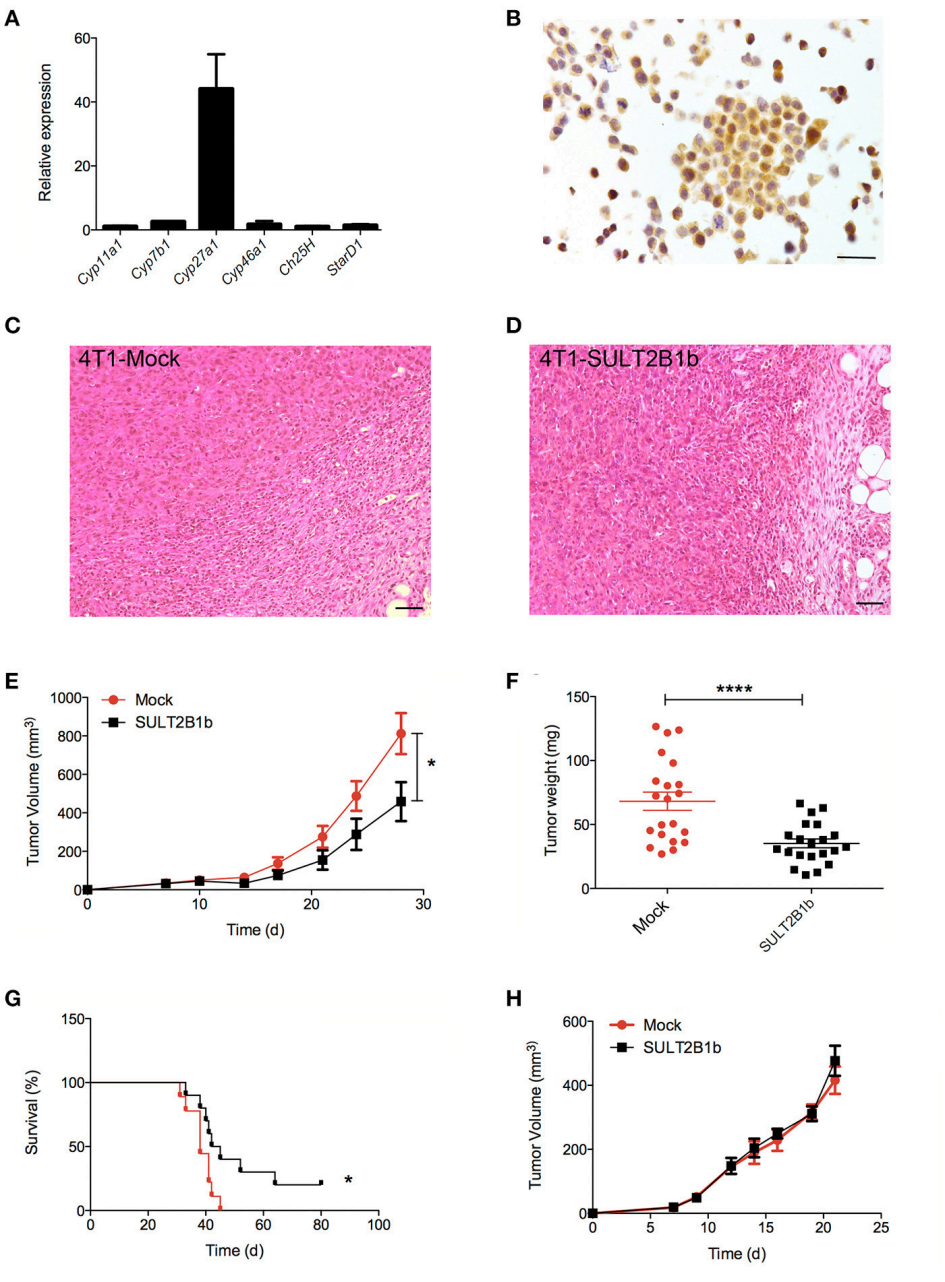
Analysis of the effects of SULT2B1b expression on the growth of 4T1 primary breast tumors. **(A)** *In vitro* qPCR analysis evaluating the expression of cholesterol hydroxylase and *StarD1* transcripts in 4T1 cells. *StarD1* encodes a transport protein that regulates cholesterol transfer within the mitochondria. **(B)** Expression of Cyp27a1 protein by 4T1 cells. The analysis was performed by immunohistochemistry using an anti-Cyp27a1 antibody. Original magnification 400x; bar, 100 μm. **(C,D)** H&E analysis of Mock-4T1 and SULT2B1b-4T1 tumors. At tumor-host interface it is visible a less dense inflammatory infiltrate in SULT2B1b-4T1 tumors. Original magnification 200x; bar, 100 μm. **(E)** Mock-4T1 and SULT2B1b-4T1 tumor growth in BALB/C mice. Tumor cells were implanted orthotopically (*n* = 7 mice/group). **(F)** Tumor weight 14 days after tumor challenge. **(G)** Kaplan-Meier survival analysis of mice bearing Mock-4T1 and SULT2B1b-4T1 tumors (*n* = 10 mice/group). **(H)** Mock-4T1 and SULT2B1b-4T1 tumor growth in immunodeficient NOD-SCID mice (*n* = 4 mice/group). *P < 0.05; ****p < 0.0001 [Student's *t*-test and Log-rank (Mantel-Cox) test for survival experiments].

Our group has described how tumor-derived oxysterols negatively condition tumor-infiltrating immune cells, thus promoting tumor growth (13). To investigate whether and how tumor-derived oxysterols were also able to regulate the metastatic process, we selected a breast tumor model, i.e., the 4T1 cell line, which spontaneously gives rise to lung metastases after an initial growth when transplanted orthotopically in the mammary fat pad of BALB/C mice. 4T1 cells express *Cyp27a1* transcripts (Figure 1A), which encode the enzyme responsible for the generation of the oxysterol 27-Hydroxycholesterol (27-HC), and Cyp27a1 protein (Figure 1B), as described in other breast cancer models (15). To deplete 4T1 cells of active oxysterols we took advantage of the oxysterol-inactivating enzyme sulfotransferase 2B1b (SULT2B1b), a strategy successfully used by our group with other tumor models (18, 19). We therefore transduced 4T1 cell line with a lentivirus encoding the SULT2B1b (19), which converts oxysterols in their sulfated, inactive forms, thus blocking their capacity to bind the Liver X Receptors (LXRs). As control, we transduced the 4T1 cell line with a mock lentivirus (19). When injected orthotopically, both cell lines formed palpable tumors starting from 7 days after injection (Figures 1C,D). However, SULT2B1b-trasduced tumors showed a significant growth delay both in terms of volumes and weights (Figures 1E,F). This led to a statistically significant increase of the overall survival of 4T1-SULT2B1b tumor-bearing mice (Figure 1G). *In vitro* proliferation and Annexin V/PI assays ruled out the possibility that the delayed tumor growth could be the result of intrinsic defects induced by the activity of SULT2B1b enzyme (Supplementary Figures 1A,B). Tumor growth delay relied on the *in vivo* interaction between tumor cells and an intact immune system, as demonstrated by tumor challenge experiments in immune-compromised NOD-SCID mice, showing similar rates of growth of SULT2B1b- and Mock-4T1 tumors (Figure 1H).

### Sulfotransferase 2B1b-mediated oxysterol depletion in 4T1 tumor cells influences the TME composition

**Figure 2:**
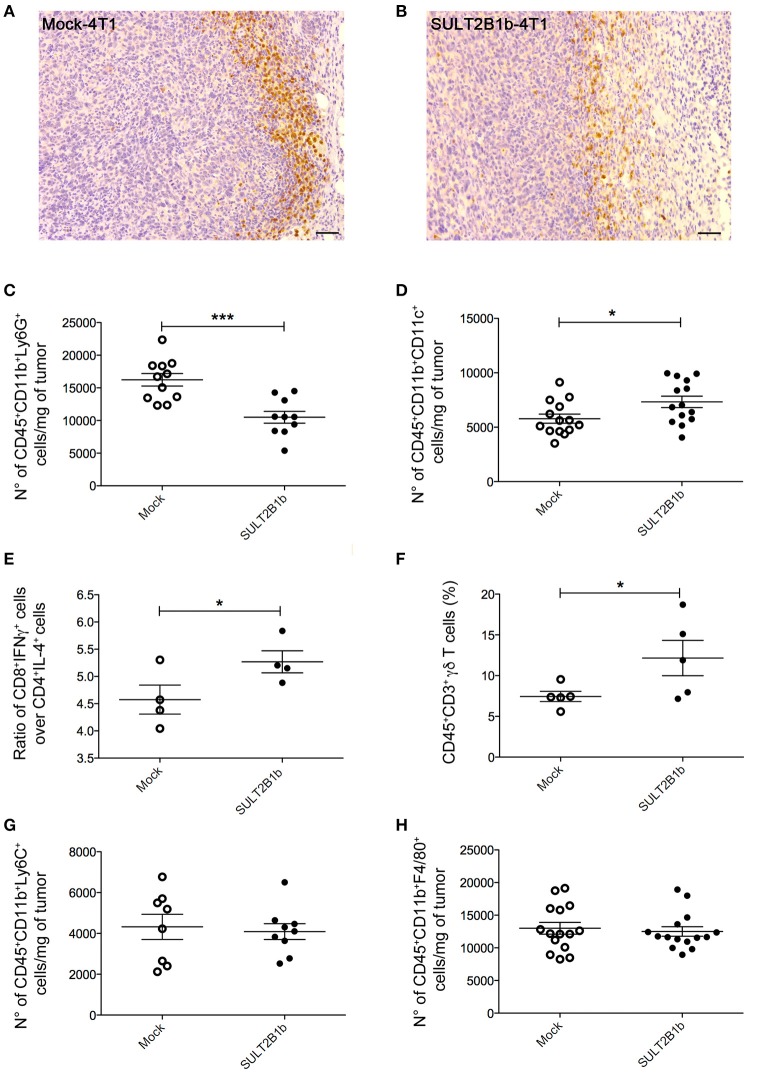
TME modifications in the presence of SULT2B1b-4T1 tumors. **(A,B)** Tumor-host interface of Mock-4T1 **(A)** and SULT2B1b-4T1 **(B)** primary breast tumors immunostained with anti-Ly6G mAb. A reduced density of Ly6G^+^ neutrophils is visible in SULT2B1b-4T1 tumors **(B)**. Original magnification 200x; bar, 100 μm. **(C,D)** Quantification of neutrophils **(C)** and dendritic cells **(D)** by flow cytometry analysis of Mock-4T1 and SULT2B1b-4T1 tumors after mechanical and enzymatic digestion. Enumeration was expressed as the number of cells/mg of tumor tissue. **(E)** Ratio between CD8^+^IFNγ^+^ and CD4^+^IL4^+^ T cells. **(F)** Percentage of CD3^+^ γδ^+^ T cells. **(G,H)** Quantification of the CD11b^+^Ly6C^+^ **(G)** and CD11b^+^F4/80^+^ **(H)** cells by flow cytometry analysis of Mock-4T1 and SULT2B1b-4T1 tumors after mechanical and enzymatic digestion Enumeration was expressed as the number of cells/mg of tumor tissue. *p < 0.05. ***p < 0.001 (Student's *t*-test).

**Figure 3:**
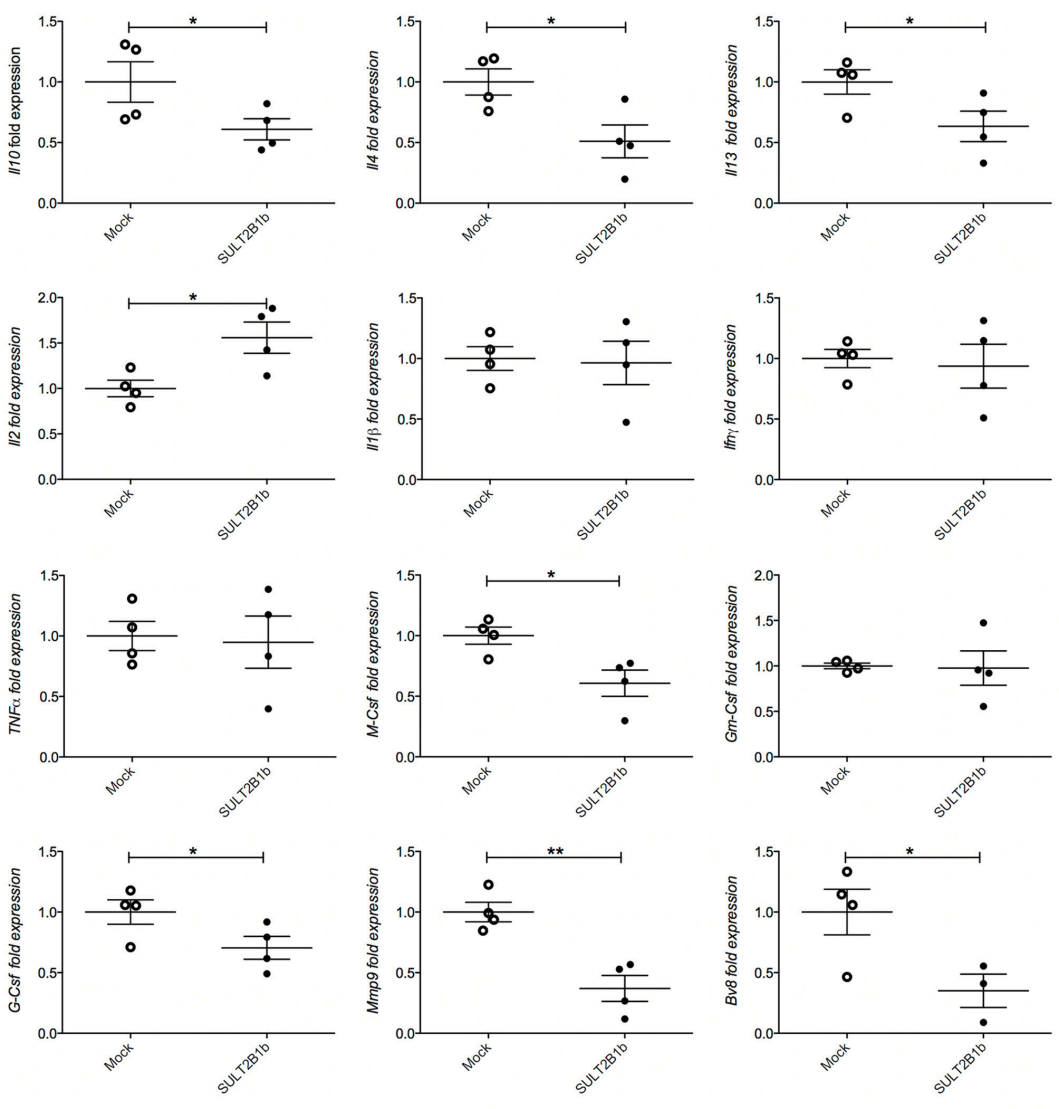
qPCR analysis of cytokines, growth factors, MMP9 and Bv8 transcripts in Mock- and SULT2B1b-4T1 tumors. Tumors were collected 7 days after orthotopic injection in BALB/C animals and subjected to RNA extraction and qPCR analysis. *p < 0.05; **p < 0.01 (Student's *t***-**test).

To evaluate whether previously identified cellular mechanisms orchestrated by tumor-derived oxysterols (18, 19) were responsible for the delay of SULT2B1b-4T1 growth *in vivo*, we performed extensive flow cytometry analyses to characterize and compare the tumor microenvironment (TME) composition of SULT2B1b- and Mock-4T1 tumors. In accordance with our previous work (18, 19), we observed a reduction of Ly6G^+^ neutrophils in SULT2B1b-4T1 tumors by immunohistochemistry (Figures 2A,B). This was confirmed more accurately by flow cytometry enumerating the numbers of CD11b^+^Ly6G^+^neutrophils/mg of tumor tissue (Figure 2C). Moreover, we detected increased numbers of F4/80^−^CD11b^+^CD11c^+^ dendritic cells in SULT2B1b-4T1 tumors (Figure 2D). These cells are critically involved in tipping the balance of the TME. In particular, low numbers of neutrophils and high numbers of DCs have been shown to convert the immune suppressive or pro-tumor TME toward a TME endowed with an antitumor ability (20). We then analyzed the adaptive component of TME and we did not observe a significant modification of CD3^+^, CD8^+^, or CD4^+^ T-cell numbers between the two tumors (Supplementary Figures 2A–C). However, a more in-depth analysis showed a difference in the ratio between CD8^+^IFNγ^+^ T cells and CD4^+^IL4^+^ T cells (Figure 2E), with an increase of type 1 T-cells in the SULT2B1b-4T1 TME. We also observed an increased percentage of γδ T cells and a decreased percentage of CD8^+^PD1^+^ T-cells in SULT2B1b-4T1 tumors (Figure 2F and Supplementary Figure 2D, respectively**)**. CD4^+^FoxP3^+^ T_reg_ cells were equally represented in both tumors (Supplementary Figure 2E). Furthermore, we did not detect different numbers of monocytes (CD11b^+^Ly6C^+^ cells) and macrophages (CD11b^+^F4/80^+^ cells) infiltrating the two tumors (Figures 2G,H). To gain insights into the different functional activity of these cells, we purified CD11b^+^ cells, also including macrophages, from SULT2B1b- and Mock-4T1 tumors and analyzed by qPCR the prototypic genes discriminating between type 1 and type 2 cells. However, we failed to observe any significant diversity in terms of cell polarization between SULT2B1b- and Mock-4T1 tumors (Supplementary Figure 3). Overall, these results indicate that the depletion of active oxysterols through the SULT2B1b enzyme deeply affects the cellular components of the TME, tipping the balance toward a TME made up of immune cells bearing phenotypic features potentially responsible for antitumor responses. This was confirmed by the analysis of cytokine transcripts present in the growing tumors (Figure 3). We observed reduced levels of transcripts encoding anti-inflammatory cytokines, such as *Il10, Il4*, and *Il13* in SULT2B1b-4T1 tumors. Additionally, we found higher levels of *Il2* and lower levels of *m-csf*, *g-csf* and *mmp9* in SULT2B1b-4T1 tumors, the latter possibly correlating with the lower number of neutrophils infiltrating SULT2B1b-4T1 tumors (Figures 3, 2C). In accordance with this hypothesis, we detected decreased transcript levels of the neutrophil-secreted proangiogenic factor *Bv8* (Figure 3). Unexpectedly, we failed to detect a difference in terms of *Ifn*γ transcripts.

### Role of oxysterols in 4T1 metastasis formation

**Figure 4:**
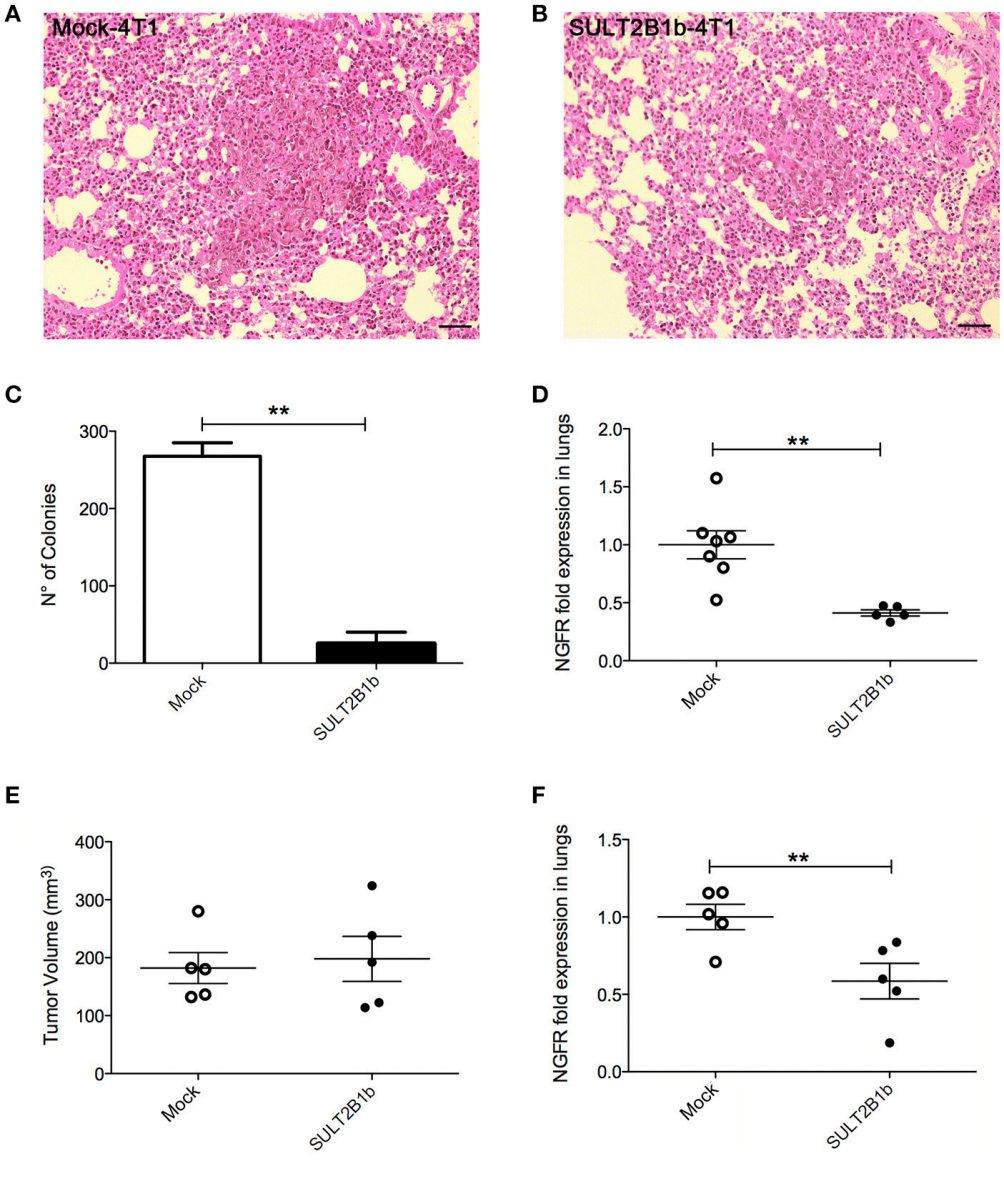
SULT2B1b expression affects *in vivo* metastatic behaviour of 4T1 tumor cells. **(A,B)** H&E analysis of Mock-4T1 and SULT2B1b-4T1 lung metastatic nodules. The inflammatory component surrounding the SULT2B1b-4T1 lung metastatic nodule **(B)** is less dense than that surrounding the Mock-4T1 lung metastatic nodule **(A)**. Original magnification 200x; bar, 100 μm. **(C)** Clonogenic assay and **(D)** qPCR analysis of lungs of tumor-bearing mice. **(E)** Primary tumor growth and **(F)** qPCR analysis of lungs of tumor-bearing mice, in which Mock-4T1 and SULT2B1b-4T1 primary tumors were surgically removed after reaching the same size. **p < 0.01. (Student's *t*-test).

The decreased overall survival observed in Mock-4T1 tumor-bearing mice (Figure 1D) led us to hypothesize a biological role of oxysterols also in the metastatic process. Since the orthotopic injection of 4T1 breast tumor cells gives rise to lung metastatses (17), we analyzed and detected by histology metastatic nodules in lungs of Mock-4T1- and SULT2B1b-4T1-bearing mice (Figures 4A,B). To quantify the number of metastases, lungs of tumor-bearing mice were processed to obtain single cell suspensions, which were subsequently cultured for 14 days in the presence of the 6-thioguanine to select 4T1 metastatic resistant cells. We found an increased number of metastatic colonies in Mock-4T1 as compared to SULT2B1b-4T1 samples (Figure 4C). These data were confirmed by immunohistochemical analysis (*data not shown*). Exploiting the *NGFr* reporter gene encoded by both Mock and SULT2B1b lentiviruses, we devised a complementary assay using qPCR to measure the number of metastatic cells in the lungs (Figure 4D). This assay confirmed that Mock-4T1 cells were more prone to form lung metastases, as compared to SULT2B1b-4T1 cells. We also detected an increased expression of *NGFr* transcripts in the blood of Mock-4T1 tumor-bearing mice, though the results were not statistically significant (*data not shown*). We hypothesized that the decreased number of metastatic cells in the lungs could be the result of the delayed growth of SULT2B1b-4T1 primary tumors. Therefore, we modified the *in vivo* experimental design, allowing the SULT2B1b-4T1 tumors to reach sizes equal to those observed in 4T1-Mock tumors at the end-points. The analysis of lungs demonstrated that the increased capacity of Mock-4T1 tumors to give rise a higher number of lung metastases was independent of the primary tumor burden (Figures 4E,F).

The epithelial-mesenchymal transition (EMT) is a well-established process allowing epithelial tumor cells to gain migratory and invasive properties, ultimately leading to metastasis formation (21). LXR activation in tumor cells has been shown to induce the up-regulation of genes involved in the EMT (15). Therefore, we sought to investigate whether a differentially expression pattern of EMT genes was responsible for the different metastatic behaviour of Mock- and SULT2B1b-4T1 cells. However, epithelial (*mCdh1*) or mesenchymal (*mSnai1, mSnai2* and *mVIM*) genes were not differentially expressed between Mock- and SULT2B1b-4T1 cells (Supplementary Figure 4A). Furthermore, the levels of TGFβ, a protein involved in EMT, were not different in the supernatants of *in vitro* cultured Mock- and SULT2B1b-4T1 cells (Supplementary Figure 4B), or when measured *in vivo* (Supplementary Figure 4C). These results suggest that the expression of SULT2B1b does not modify the molecular pattern associated to EMT of 4T1 breast tumor cells. Instead, we observed by flow cytometry a slight decrease of β1 and β3 integrins in SULT2B1b-4T1 cells as compared to Mock-4T1 cells, whereas α5 and αv integrins were similarly expressed by the two cell lines (Supplementary Figure 4D).

### Dissecting the mechanisms responsible for the different metastatic capabilities of mock-4T1 and SULT2B1b-4T1 tumor cells

**Figure 5:**
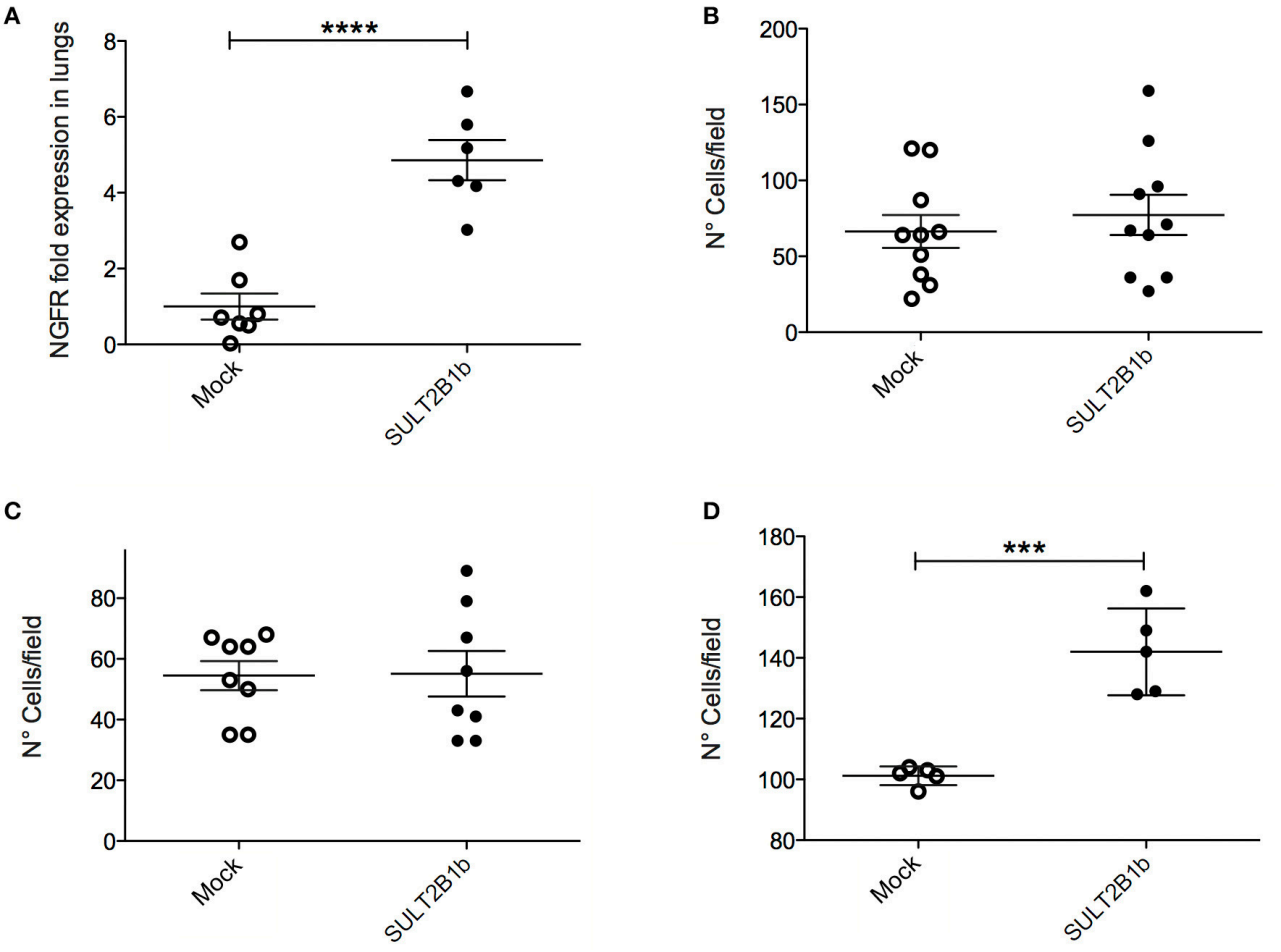
*In vivo* and *in vitro* analysis of SULT2B1b-4T1 cell metastatic capabilities. **(A)** qPCR analysis of lungs after the injection of SULT2B1b-4T1 cells in the tail vein of naive mice. **(B–D)** *in vitro* migration assays using transwell chambers untreated **(B)**, or coated with a matrigel layer **(C)**, or filled up with a monolayer of HUVEC cells left growing on top of the filter **(D)**. ***p < 0.001; ****p < 0.0001 (Student's *t*-test).

To get insights into the mechanisms of metastasis formation we performed *in vivo* metastasis formation assays. Unexpectedly, when we injected tumor cells directly into the mouse tail vein, SULT2B1b-4T1 cells colonized the lungs much more efficiently as compared to Mock-4T1 cells (Figure 5A), indicating a more pronounced attitude of SULT2B1b-4T1 cells to establish lung nodules. To better understand how oxysterols affected the metastatic process of 4T1 cells, we resorted to *in vitro* assays. We thus performed standard migration and invasion assays, where Mock- and SULT2B1b-4T1 cells were seeded on the top chamber of transwell filters without or with Matrigel, and were left to migrate to the NIH-3T3 conditioned media. No differences were found between the two cell lines (Figures 5B,C). However, when we performed the transendothelial migration assays, in which tumor cells migrate to the NIH-3T3 conditioned media by crossing a monolayer of HUVEC cells left growing on top of the filter, the SULT2B1b-4T1 cells migrated much more efficiently than Mock-4T1 cells (Figure 5D), mimicking the results obtained *in vivo* following the intravenous injection of SULT2B1b-4T1 cells (Figure 5A). These results indicate that 4T1 tumor cells expressing SULT2B1b are endowed with a higher capacity to give rise to metastases. Since this capacity is lost in the presence of established primary tumors and does not depend on the tumor size (Figures 4E,F), we hypothesized that the TME of SULT2B1b-4T1 tumors could influence the subsequent metastatic cascade of 4T1 tumor cells. *Mmp9* transcripts resulted to be significantly decreased in SULT2B1b-4T1 tumors before any difference in size could be observed (Figure 3). These results correlated with the above-reported reduction of neutrophils in the primary SULT2B1b-4T1 tumors (Figure 2C). Neutrophils have recently been associated to the formation of lung metastases in breast tumor models (17, 22). Both observations suggest a possible contribution of the primary TME to the reduced metastatic potential of SULT2B1b-4T1 tumors, independently of intrinsic tumor cell modifications induced by SULT2B1b expression and activity.

**Figure 6:**
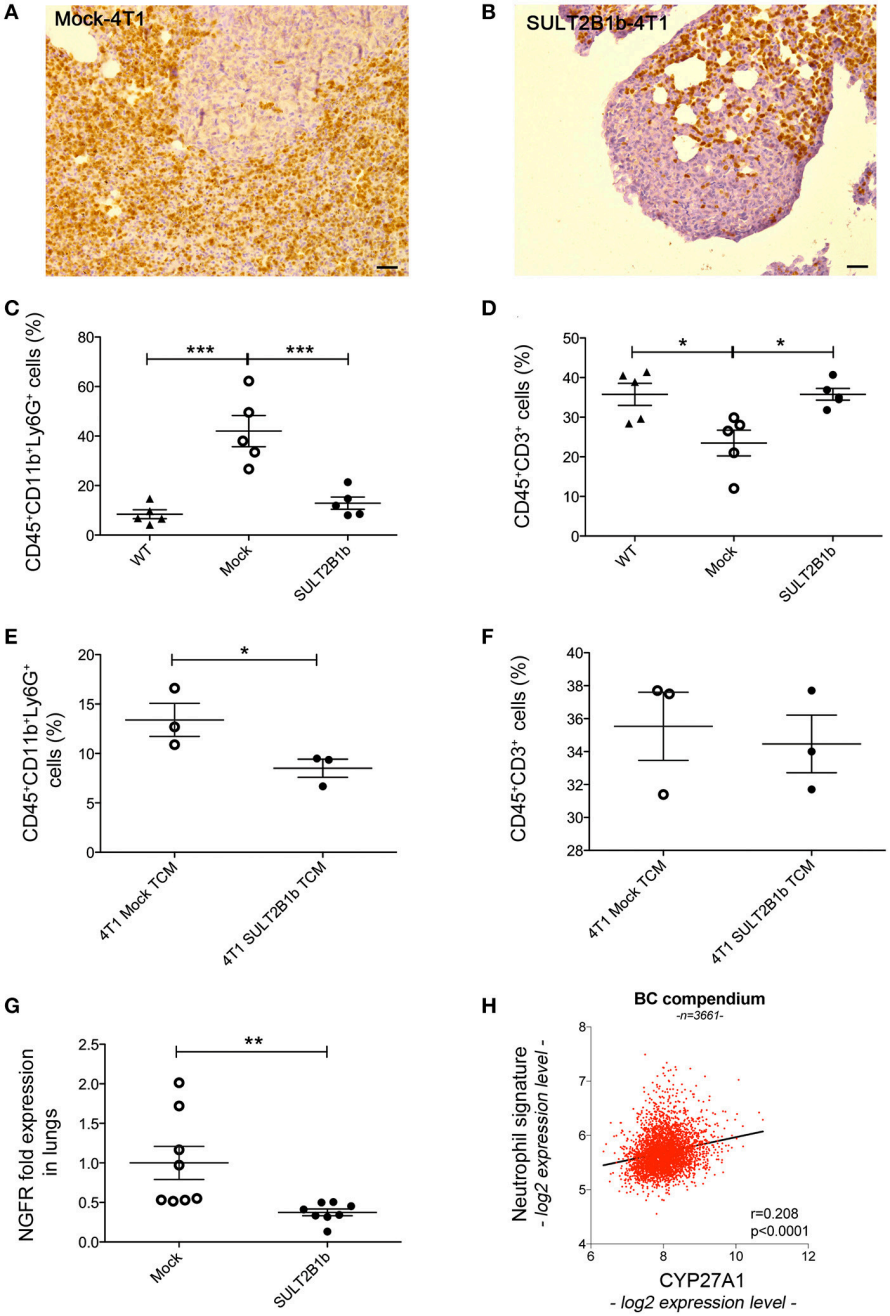
SULT2B1b expression in the primary tumors affects the lung metastatic niche. **(A,B)** Immunoistochemical analysis of Mock-4T1 **(A)** and SULT2B1b-4T1 **(B)** lung metastases immunostained with anti-Ly6G mAb. The density of Ly6G^+^ neutrophils is reduced in SULT2B1b-4T1 tumors **(B)**. Original magnification 200x; bar, 100 μm. **(C,D)** Percentage of neutrophils **(C)** and CD3^+^ T-cells **(D)** by flow cytometry analysis of naïve lungs, Mock-4T1 and SULT2B1b-4T1 lung metastases after mechanical and enzymatic digestion. *p < 0.05; ***p < 0.001 (ANOVA). **(E,F)** Percentage of neutrophils **(E)** and CD3^+^ T-cells **(F)** by flow cytometry analysis of lungs collected from naïve mice treated with tumor conditioned media (TCM) from Mock-4T1 and SULT2B1b-4T1 tumors, after mechanical and enzymatic digestion. *p < 0.05; (Student's *t*-test). **(G)** qPCR analysis for NGFR expression of lungs from Mock-4T1 and SULT2B1b-4T1 tumor-bearing NOD-SCID mice. **p < 0.01. (Student's *t* test). **(H)** Scatter plot (red dots) and linear regression (black line, slope 0.532) of expression values indicate a positive correlation between the expression level of *CYP27A1* and a neutrophil gene signature, in a compendium of 3661 primary human breast cancers (see Materials and Methods). The Pearson ρ quantifies the linear dependence between the expression levels of *CYP27A1* and of the neutrophil signature (*p*-value < 0.0001).

The above-reported results led us to hypothesize that the TME of the primary tumors could be able to shape the environment of the lung metastatic niche. Immunohistochemistry with anti-Ly6G mAb of lungs from tumor-bearing mice showed a high neutrophil infiltration of metastatic lungs from Mock-4T1- and SULT2B1b-4T1-bearing mice (Figures 6A,B). Similar immunohistochemical features were also observed in the lung interstitium from tumor-bearing mice (*data not shown*). Immunohistochemical analyses were confirmed by FACS analysis performed on the lungs of naïve and tumor-bearing mice. We observed an increase of CD11b^+^Ly6G^+^ neutrophils in the lungs of Mock-4T1 tumor-bearing mice as compared to both naïve and SULT2B1b-4T1 tumor-bearing mice (Figure 6C). On the other hand, CD3^+^ T cells were decreased in the lungs of Mock-4T1 tumor-bearing mice as compared to both naïve and SULT2B1b-4T1 tumor-bearing mice (Figure 6D). An in-depth analysis showed a slight difference of the percentage of CD3^+^CD4^+^ and CD3^+^CD8^+^ T cells in the lungs of Mock-4T1 and SULT2B1b-4T1-tumor-bearing mice, with a lower percentage and a higher percentage of tumor-infiltrating CD3^+^CD4^+^ and CD3^+^CD8^+^ T cells, respectively, in the lungs of SULT2B1b-4T1-bearing mice (Supplementary Figures 5A-C). The percentage of CD4^+^IL4^+^ T cells and CD8^+^IFNγ^+^ T cells infiltrating the lung metastases of Mock-4T1 and SULT2B1b-4T1-tumor-bearing mice was extremely low and no difference between the two groups was observed (Supplementary Figures 5D,E). Differently from the primary tumors, we did not observe any difference in the percentage of γδ T cells between the two groups (Supplementary Figure 5F). Moreover, lungs of SULT2B1b-4T1 tumor-bearing mice showed a decrease of CD11b^+^Ly6C^+^ monocytes (Supplementary Figure 6A) and an increase of CD11b^low^CD11c^+^ tissue resident macrophages (Supplementary Figure 6B) (23), as compared to both naïve (WT) mice and Mock-4T1 tumor-bearing mice. Finally, CD11b^+^F4/80^+^ macrophages decreased in the lungs of Mock and SULT2B1b tumor-bearing mice as compared to naive (WT) mice (Supplementary Figure 6C). Similar results could be obtained by treating naïve mice intraperitoneally with the conditioned media (CM) of Mock- and SULT2B1b-4T1 cells. Mice received 300 μl of CM intraperitoneally every day for 2 weeks. Lungs were then recovered and analyzed by flow cytometry. We observed a different percentage of neutrophils and macrophages in the lungs of naïve mice treated with SULT2B1b-4T1 tumor CM (Figure 6E and Supplementary Figure 6F), while other immune cells did not undergo any modifications (Supplementary Figures 6D,E). Moreover, we did not detect any significant difference of CD3^+^ T cells infiltrating lungs of Mock- and SULT2B1b-4T1 bearing mice (Figure 6F).

Altogether these results suggest that in the 4T1 model the enzymatic depletion of oxysterols in the primary tumors, modulates the formation of lung metastases by regulating the levels of immune cells infiltrating the metastatic TME. Indeed, lung metastases of SULT2B1b-4T1 tumors were infiltrated by lower numbers of tumor-promoting neutrophils and higher numbers of CD3^+^ T cells, though the latter were not confirmed by the intraperitoneal injections of CM of Mock- and SULT2B1b-4T1 cells. To finally prove whether neutrophils, T cells or both subsets were involved in lung metastasis formation, we performed tumor challenge experiments in immunodeficient NOD-SCID mice, which lack lymphocytes. Although the primary Mock- and SULT2B1b-4T1 primary tumors grew similarly in immunodeficient NOD-SCID mice (Figure 1H), the number of metastases in the lungs of SULT2B1b-4T1 tumor-bearing mice was significantly lower as compared to lungs of Mock-4T1 tumor-bearing mice (Figure 6G). These results strongly suggest that neutrophils would represent the main drivers of lung metastatic formation in the 4T1 breast tumor model.

Finally, to identify a possible correlation between increased tumor-infiltrating neutrophils and oxysterol content in human breast tumors, we interrogated 3661 primary breast tumor samples, and we found a significant direct correlation between the expression of transcripts identifying a neutrophil signature and the expression of transcripts for *CYP27A1*, which encodes the oxysterol 27-HC (*r* = 0.208; *p* < 0.0001) (Figure 6H).

## Discussion

Here, we have investigated the contribution of the LXR/oxysterol axis to the formation of primary and metastatic breast tumors. To this aim we took advantage of the sulfotransferases 2B1b (SULT2B1b), which inactivates oxysterols through the sulfation of hydroxyl groups (24). In line with our previous results (19, 25), the injection of 4T1 breast tumor cells, genetically engineered to express SULT2B1b, resulted in tumor growth delay and prolonged overall survival (Figures 1E–G), indicating that oxysterols contribute to the establishment of primary 4T1 breast tumors by impinging on antitumor immune responses, as demonstrated by experiments carried out in immunodeficient mice (Figure 1H). Neutrophils have been shown to play a dual role within the TME, as they evolve from anti-tumour to pro-tumour cells in mice bearing transplantable tumor cell lines (26). Particularly, in the TME of 4T1 growing tumors, neutrophils have been reported to have contrasting roles. Li et al. showed that the depletion of CD11b^+^Gr1^+^ neutrophils decreased the pro-tumorigenic effect of TGF-β, whereas Granot et al. reported no effects in the growing tumors (27, 28). These different outcomes could be dependent on the timing of neutrophil depletion (26). We have shown that oxysterols released from tumor cells recruit neutrophils endowed with pro-angiogenic and immunosuppressive abilities through a mechanism requiring the CXCR2 chemokine receptor and independently of LXRs (18, 25). This mechanism could be also responsible for the different number of neutrophils detected in Mock- and SULT2B1b-4T1 tumors, with a lower number of CD11b^+^Ly6G^+^ neutrophils in tumors inactivating oxysterols by means of the SULT2B1b enzymatic activity. Extensive flow cytometry analysis of the TME of SULT2B1b-4T1 and Mock-4T1 tumors, highlighted other cell differences characterizing these tumors. The ratio between effector CD8^+^IFNγ^+^ and CD4^+^IL-4^+^ T cells, the latter commonly endowed with a pro-tumor ability (29), was tipped in favour of effector cells in the SULT2B1b-4T1 TME. This observation is in accordance with the consolidated notion that tumor growth control in immunocompetent mice is frequently achieved through a T-cell response mediated by CD8^+^IFNγ^+^ effector T cells. These results are corroborated by tumor challenge experiments in NOD-SCID mice, in which we failed to observe the control of SULT2B1b-4T1 tumor growth. On the other hand, these results indicate that tumor-derived oxysterols may shape the TME inducing a pro-tumor milieu, as confirmed by the detection in the TME of Mock-4T1 tumors of higher levels of the type-2 cytokine transcripts *Il4, Il13*, as well as the anti-inflammatory *Il10* transcripts (30) (Figure 3). Of note, the tumor-promoting T helper type 2 inflammation has been reported in human breast tumor samples (31) and in human pancreatic cancer, whereby it is associated to reduced overall survival (32). *Ifn*γ transcripts were not affected by oxysterol inactivation. However, we cannot rule out a distinct mechanism of regulation of IFN-γ post-transcriptionally or at protein level. Although these results are partly in accordance with data reported in Figure 2E, additional levels of regulation may affect the expression of cytokines and chemokines, including post-transcriptional regulations. Therefore, these results deserve further investigations at protein level to get conclusive results conclude on the cytokines, chemokines and growth factors released within 4T1-Mock and 4T1-SULT2B1b TMEs.

SULT2B1b-4T1 tumors also contained a higher number of gamma delta (γδ) T-cells, an observation suggesting an antitumor role played by these cells in our experimental setting. The role of γδ T cells in the TME is greatly debated. γδ T cells were shown to efficiently kill tumor cells by releasing perforins, granzymes and IFNγ (33). Furthermore, the adoptive transfer of γδ T cells in 4T1 tumor-bearing mice has proven to be effective in contrasting the tumor growth (34, 35). On the other hand, Coffelt et al. have recently demonstrated that IL-17 released by γδ T cells induces the expansion of neutrophils in mice bearing mammary tumors, which in turn suppress the antitumor CD8^+^ T-cells (36). Further investigations are therefore needed to define stimuli and conditions associated to the TME and making γδ T cells antitumor or protumor.

4T1 tumors metastasize spontaneously to the lungs when injected orthotopically. This feature makes this model useful to investigate the metastatic process. Since SULT2B1b-4T1 tumor bearing mice showed a lower number of lung metastases (Figure 4), we asked how LXR/oxysterol axis influenced the metastatic capacity of 4T1 tumors. Nelson et al. have shown that LXR engagement increases the metastatic spreading of breast tumor cells by activating the EMT pathway (15). However, the intrinsic role of LXR/oxysterol axis to induce lung metastases was ruled out by the evidence that some transcripts characterizing the EMT phenotype were similarly expressed by Mock- and SULT2B1b-4T1 cells (Supplementary Figure 4A). Moreover, we did not observe any difference in the levels of TGFβ, a key mediator of EMT (21) between SULT2B1b-4T1 and Mock-4T1 tumors, both *in vitro* and *ex vivo*. In contrast to the *in vivo* experiments, SULT2B1b-4T1 tumor cells showed a higher capability to form lung metastases when injected i.v. (Figure 5A), and to migrate across the endothelium when tested *in vitro* by transendothelial migration assays (Figure 5D). We then asked whether the cellular and molecular modifications induced by tumor-derived oxysterols in the primary tumor sites were also impacting on the metastatic cascade. Neutrophils, for example, through the secretion of MMP9 and G-CSF, favour the metastatic capacity of tumor cells (37, 38). In agreement with these results, we found a higher percentage of neutrophils in the metastatic lungs of Mock-4T1 mice, suggesting a possible role played by oxysterols in recruiting immunosuppressive and pro-tumor neutrophils in the metastatic lungs. This was further supported by the experiments in NOD-SCID mice, where an increased number of metastatic cells could be detected in Mock-4T1 tumor-bearing mice regardless of the absence of T cells. The role of neutrophils in conditioning the metastatic niche is still debated and might be dependent on the complex molecular and cellular networks occurring in the TME (26). Indeed, some groups have shown that neutrophils actively kill early metastatic cells through the release of H_2_O_2_ (28). Other groups have instead reported the pro-tumor role exerted by neutrophils in promoting metastasis formation through the decrease of IFNγ production, the secretion of the vasculature remodelling protein MMP9 and the secretion of neutrophil-derived leukotrienes (17, 22). Factors released by the growing tumors are indeed able to induce such an early recruitment of neutrophils in the lungs, as suggested by our experiments with conditioned media from Mock-4T1 tumors (Figure 6E).

Overall, these results demonstrate that oxysterols depletion through the enzymatic activity of SULT2B1b reprograms the tumor microenvironment favouring the control of breast tumors and metastasis formation. Neutrophils are primarily involved in this mechanism, though we cannot rule out a possible pro-metastatic role exerted by CD11b^+^F4/80^+^ cells, which we found decreased in the lungs of mice injected with conditioned media of SULT2B1b-4T1 tumor cells. Finally, a work recently published by Baek et al. (39) demonstrates that the oxysterol 27-HC plays a pro-metastatic role in breast cancer models through the recruitment of immune suppressive neutrophils in the metastatic niche.

Our work opens new therapeutic avenues for the prevention of the metastatic disease in breast cancer patients through strategies counteracting oxysterol generation. This observation is also supported by experiments reported in Figure 6H and demonstrating a positive correlation between tumor-infiltrating neutrophils and the expression of the CYP27A1 enzyme generating the oxysterol 27-HC.

## Materials and methods

### Cell lines and reagents

4T1 mammary tumor cell line was cultured in RPMI 1640 (Roswell Park Memorial Institute) complemented with 10% fetal bovine serum (FBS, Euroclone Ltd.), 2mM L-Glutammine (GIBCO) and 1% penicillin/streptomycin (GIBCO). 4T1-SULT2B1b and 4T1-Mock tumor cells were obtained by engineering 4T1 cells with lentivirus encoding SULT2B1b or empty vector encoding only the ΔNGFr cell surface marker, as described previously Villablanca et al. (19). Analysis of mRNA and flow cytometry were performed when cells were in an exponential growth phase. Reverse transcription was performed by incubating 1–2 μg of total RNA [extracted using Trizol reagent (Invitrogen) according to manufacturer's protocol], with MLV-Reverse Transcriptase (Promega) for 1 h at 42°C. Quantitative PCR was performed using real-time PCR (Viia7, Applied Biosystems) using SyberGreen technology (see Table 1 for the list of qPCR primers). Cell staining was performed by incubating the cells with specific and control antibodies at 4°C for 20 min. The following antibodies were used: anti-CD45 (30-F11), -CD11b (M1/70), -CD11c (N418), -Ly6G (1A8), -Ly6C (HK1.4), -F4/80 (BM8), -CD3 (145-2C11), -CD4 (GK1.5), -CD8 (53-6.7), -PD1 (RPM1-30), -TCRγδ (GL3), -IFNγ (XMG1.2), and -IL-4 (11B11). These antibodies were all from Biolegend. Anti-FoxP3 (FJK-16s) was from eBiosceince. Anti-mouse/rat alpha5 (CD49e, clone HMα5-1), anti-mouse alphaV Integrin (CD51, clone RMV-7), anti-mouse/rat integrin beta3 (CD61 clone 2C9.G3) and anti-mouse integrin beta1 (CD29 clone HMβ1-1) were from Immunological Sciences and were a kind gift of Dr. Flavio Curnis. Anti-Cyp27a1 rabbit antibody (Clone EPR7529) was from Abcam.

**Table 1:** List of qPCR primers.

	**Forward primer**	**Reverse primer**
Rpl13	TCAAGGTTGTTCGGCTGAAG	GCCCCAGGTAAGCAAACTTT
Csf1	TCAACAGAGCAACCAAACCA	ACCCAGTTAGTGCCCAGTGA
Csf2	CTGTCACGTTGAATGAAGAGGTAG	AGCTGGCTGTCATGTTCAAGG
Csf3	GAGCAGTTGTGTGCCACCTA	GCTTAGGCACTGTGTCTGCTG
Mmp9	CAATCCTTGCAATGTGGATG	TAAGGAAGGGGCCCTGTAAT
Il10	CTGGACAACATACTGCTAACCG	GGGCATCACTTCTACCAGGTAA
Il1β	GCAACTGTTCCTGAACTCAACT	ATCTTTTGGGGTCCGTCAACT
Il2	TGAGCAGGATGGAGAATTACAGG	GTCCAAGTTCATCTTCTAGGCAC
Ifnγ	ATGAACGCTACACACTGCATC	CCATCCTTTTGCCAGTTCCTC
Il4	ACTTGAGAGAGATCATCGGCA	AGCTCCATGAGAACACTAGAGTT
Il13	AGACCAGACTCCCCTGTGCA	TGGGTCCTGTAGATGGCATTG
Cox-2	TGAGCAACTATTCCAAACCAGC	GCACGTAGTCTTCGATCACTATC
Tnfα	AGGGATGAGAAGTTCCCAAATG	CACTTGGTGGTTTGCTACGAC
iNos	GTTCTCAGCCCAACAATACAAGA	GTGGACGGGTCGATGTCAC
Arg1	CCACAGTCTGGCAGTTGGAAG	GGTTGTCAGGGGAGTGTTGATG
Mgl1	CAGAATCGCTTAGCCAATGTGG	TCCCAGTCCGTGTCCGAAC
Fizz1	CCTGCTGGGATGACTGCTA	TGGGTTCTCCACCTCTTCAT
Mrc1	AAGGCTATCCTGGTGGAAGAA	AGGGAAGGGTCAGTCTGTGTT
NGFR	ACAAGACCTCATAGCCAGCA	CCCCTGTTCCACCTCTTGAA
Cdh1	CAGGTCTCCTCATGGCTTTGC	CTTCCGAAAAGAAGGCTGTCC
Snai1	CACACGCTGCCTTGTGTCT	GGTCAGCAAAAGCACGGTT
Snai2	TGGTCAAGAAACATTTCAACGCC	GGTGAGGATCTCTGGTTTTGGTA
Vim	CGTCCACACGCACCTACAG	GGGGGATGAGGAATAGAGGCT
Cyp11a1	AACATCCAGGCCAACATTAC	GGTCATGGAGGTCGTGTC
Cyp27a1	GACCTCCAGGTGCTGAAC	CTCCTGTCTCATCACTTGCTC
Cyp46a1	GTGTGCTCCAAGATGTGTTTCT	ACACAGTCTGAAGCGCGCGGT
Cyp7b1	TCAGGAAAGGCAAGATCTGCTGA	CCTGTTGACTGCAGGAAACTGTCA
Lxrα	AGGAGTGTCGACTTCGCAAA	CTCTTCTTGCCGCTTCAGTTT
Lxrβ	AAGCAGGTGCCAGGGTTCT	TGCATTCTGTCTCGTGGTTGT

### *In vitro* proliferation and apoptosis assays

For the proliferation assay, 20.000 cells were plated in triplicate in 24-well plates and cultured at 37°C. At the indicated time point the cells were collected and counted by trypan blue exclusion. The Annexin V-PI assay (apoptosis assay) was performed according to the manufacturer's instructions using the Annexin V-FITC detection kit I (BD Biosciences). Cell pellets were resuspended in 100 μl of binding buffer and incubated with 5 μl of FITC-conjugated Annexin-V and 5 μl of PI for 15 min at room temperature in the dark. 400 μl of 1 × binding buffer was added and the samples were immediately analyzed by flow cytometry. Samples were run on a Canto II flow cytometer (BD) and analyzed by FlowJo software, gating on live cells.

### Tumor challenge experiments

For orthotopic experiments, cells were washed three times with PBS without calcium and magnesium (PBS-). Cells were then injected in the mammary fat pad of BALB/C mice or NOD-SCID mice, and tumor volume was determined by measuring three perpendicular axes with a caliper starting from day 7 after injection. In some experiments, 30.000 Mock- or SULT2B1b-4T1 cells were injected in the tail vein of BALB/C mice. After 14 days, lungs were recovered and analyzed for NGFr expression as reported above. Animal studies were approved by the Institutional Animal Care and Use Committee of the San Raffaele Scientific Institute (IACUC n° 656). All the experiments *in vivo* were performed by using BALB/C mice, with exception of the experiments reported in Figures 1H, 6G, whereby we used NOD-SCID mice.

### Immunohistochemistry

Heat-induced antigen retrieval in Tris-EDTA buffer (pH 9.0) for 30 min at 97 °C was used, followed by blocking of endogenous peroxidase with 3% H_2_O_2_ and incubation with 3% of normal bovine serum. Ly6G (1A8 clone; BioLegend) primary antibody was incubated for 1 h at RT, followed by detection with rat on mouse HRP conjugated-polymer (RT 517, Biocare Thermo Scientific, Fremont, CA, USA) and developed with DAB chromogen. Tissue sections were counterstained with hematoxylin and evaluated with a Nikon i80 microscope.

### Analysis of tumor-infiltrating immune cells

Tumors were recovered and weighted at day 14 of growth, then digested using a mix of Collagenase A, B and D (final concentration 1,7 mg/ml; Roche) for 30 min at 37°C. Digested samples were passed through a 70 μm cell strainer, washed and counted. Cells were then stained were stained with Live/Dead Staining (Life Technologies), then with a mix of antibodies (Biolegend) according to manufacturer's instructions and analyzed by flow cytometry. For gating strategy see Supplementary Figures 7, 8. For intracellular staining, cells were incubated in RPMI 10% FBS with PMA 100 ng/ml, ionomycin 500 ng/ml and brefeldin 10 μg/ml (Sigma-Aldricht) for 3 h al 37°C. Then, cells were stained with Live/Dead Staining (Life Technologies) and specific or control antibodies. Cells were finally permeabilized using Perm/wash kit (BD Biosciences) and stained with relevant intracellular antibodies. Intracellular FoxP3 staining was performed using Foxp3/Transcription Factor Staining Buffer Set (eBioscience). mRNA analysis was performed on homogenized tumors using TRIZOL reagent (Invitrogen), as reported before. In some experiments, mRNA analysis was performed on CD11b purified cells. Purification was carried out with anti-PE microbeads and Miltenyi kit according to manufacturer's instructions. CD11b isolation purity was >95% and total RNA was isolated through RNeasy Qiagen Kit, then reverse transcription was performed as reported above.

### qPCR analysis of transcripts encoding cytokines, growth factors and pro-angiogenic factors in mock- and SULT2B1b-4T1 primary breast tumors

Tumors were recovered after 7 days (before any significant difference in weight could be observed) and total RNA was extracted using Trizol reagent (Invitrogen) according to manufacturer's protocol. Reverse transcription and quantitative PCR analysis was performed as reported above.

### Analysis of lung metastasis

In the clonogenic assay, 7.000 Mock- or SULT2B1b-4T1 cells were orthotopically injected in the mammary fat pad of BALB/C mice (40). Lungs were recovered after 28 days of tumor growth and washed twice in PBS, then digested by the collagenase mix for 75 min at 4°C on a rotating wheel. Cell suspension was passed through a 70 μm cell strainer and washed once with RPMI 10% FBS. Cells were then seeded in 6-well plates at increasing serial dilutions in the presence of 60 μM 6-thyoguanine (Sigma-Aldrich). After 2 weeks of culture, the colonies were manually counted at the lowest dilution.

A complementary assay was designed to quantify the metastatic cells in the lungs and blood, using qPCR. Primers were designed on the coding sequence of NGFr reporter gene. Lungs were directly homogenized by Trizol and mRNA was extracted and analyzed as reported above. Blood samples were incubated in RBC lysis Buffer (Biologend) according to manufacturer's instructions, then lysed by Trizol and processed as reported above.

### Migration/invasion/transendothelial migration assays

For the migration assay, Mock- or SULT2B1b-4T1 cells were starved overnight, then cells were detached and counted. 30.000 cells were seeded in the top chamber of 8 μm transwell insert (Millipore) in RPMI complemented with 0.5% BSA and left to migrate toward NIH-3T3 conditioned media for 8 h. Cells in the upper part of the filters were removed with a cotton swab, then the filters were fixed in 70% ethanol for 10 min. A cotton swab was used again to remove ethanol in excess and the filters were left to air dry for 15 min. Filters were then stained with crystal violet (Sigma-Aldrich) for 10 min, then carefully washed in distilled water. Migrated cells were then counted using a light microscope. For the invasion assay, a 100 μl layer of Matrigel Growth Factor Reduced Basement Membrane Matrix, Phenol Red-Free (Corning; 0.9 mg/ml) was placed in the top chamber and left for 1 h at 37°C before seeding the cells. For the transendothelial assay, 45.000 HUVEC cells were seeded in the top chamber of transwells and cultured overnight to obtain a confluent monolayer barrier. The day after, Mock- or SULT2B1b-4T1 cells were seeded and left to migrate for 16 h, and then processed as reported above.

### Lung histological analysis

Lungs from tumor bearing mice were recovered, washed twice in PBS and fixed in formalin. Processing of the samples was performed by the Institutional Mouse Histopathology Facility.

### Analysis of lung-infiltrating immune cells

Whole lungs were recovered in order to obtain an unbiased analysis of the entire organ. Lungs from tumor-bearing mice were recovered, washed twice in PBS and digested by the collagenase mix for 75 min at 4°C on a rotating wheel. Cell suspension was passed through a 70 μm cell strainer, washed once with PBS and counted. Cells were then stained with a mix of specific and control antibodies (Biolegend) according to manufacturer's instructions and analyzed by flow cytometry. For gating strategy see Supplementary Figure 9.

### Tumor cells conditioned media preparation and administration

Mock- or SULT2B1b-4T1 cells were cultured in IMDM without FCS and phenol red (GIBCO) for 24 h. Supernatants were then collected and centrifuged to remove cell debris, aliquoted and stored at −80°C until use. Mice received 300 μl of conditioned media intraperitoneally every day for 2 weeks, then lungs were recovered and analyzed by flow cytometry as reported above.

### Collection and processing of breast cancer gene expression data

We started from 4640 breast cancer samples obtained from 27 major data sets comprising microarray data normalized and annotated with clinical information as described in Enzo et al. (41). This resulted in a breast cancer compendium comprising 3661 unique samples from 25 independent cohorts (41). The neutrophil signature is composed of the genes CXCR2, ITGAM, MPO, and FUT4 and its average expression has been calculated as the average expression of all signature genes in all the samples of the breast cancer compendium. All analyses have been conducted in R (version 3.3.2).

### Data analysis

Unless otherwise indicated, all experiments were repeated at least 3 times. Data were analyzed by one-way ANOVA followed by Tukey's multiple comparison test or Student's *t*-test using GraphPad Prism software. Data were expressed as mean ± SD, while other results were shown as mean ± SEM. P values are presented in figures as *P* < 0.05; *P* < 0.01; *P* < 0.001; or *P* < 0.0001.

## Author contributions

MM and VR designed research. MM, LR, GC, DM, MS, CD, and SB performed research. MM and VR analyzed data. MM and VR wrote the paper.

### Conflict of interest statement

The authors declare that the research was conducted in the absence of any commercial or financial relationships that could be construed as a potential conflict of interest.
