# Supplementary material for: Enzymatic Inactivation of Oxysterols in Breast Tumor Cells Constraints Metastasis Formation by Reprogramming the Metastatic Lung Microenvironment
Source: Front Immunol. 2018 Oct 2;9:2251. doi: 10.3389/fimmu.2018.02251 (PMC6176086; doi:10.3389/fimmu.2018.02251)
Supplement: Supplementary file 1 [file Presentation_1.PPTX]

## Slide 1
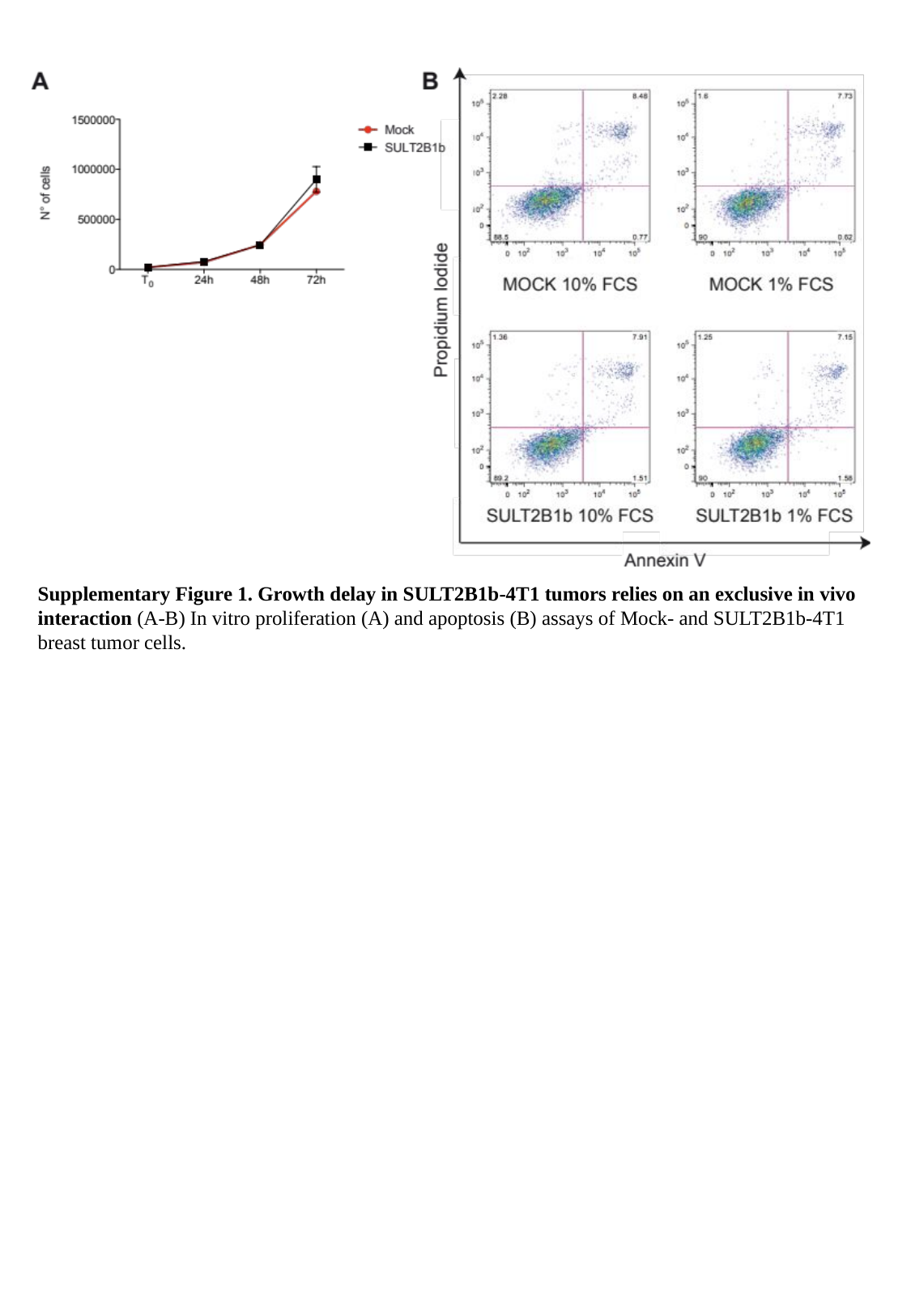

Supplementary Figure 1. Growth delay in SULT2B1b-4T1 tumors relies on an exclusive in vivo interaction (A-B) In vitro proliferation (A) and apoptosis (B) assays of Mock- and SULT2B1b-4T1 breast tumor cells.

## Slide 2
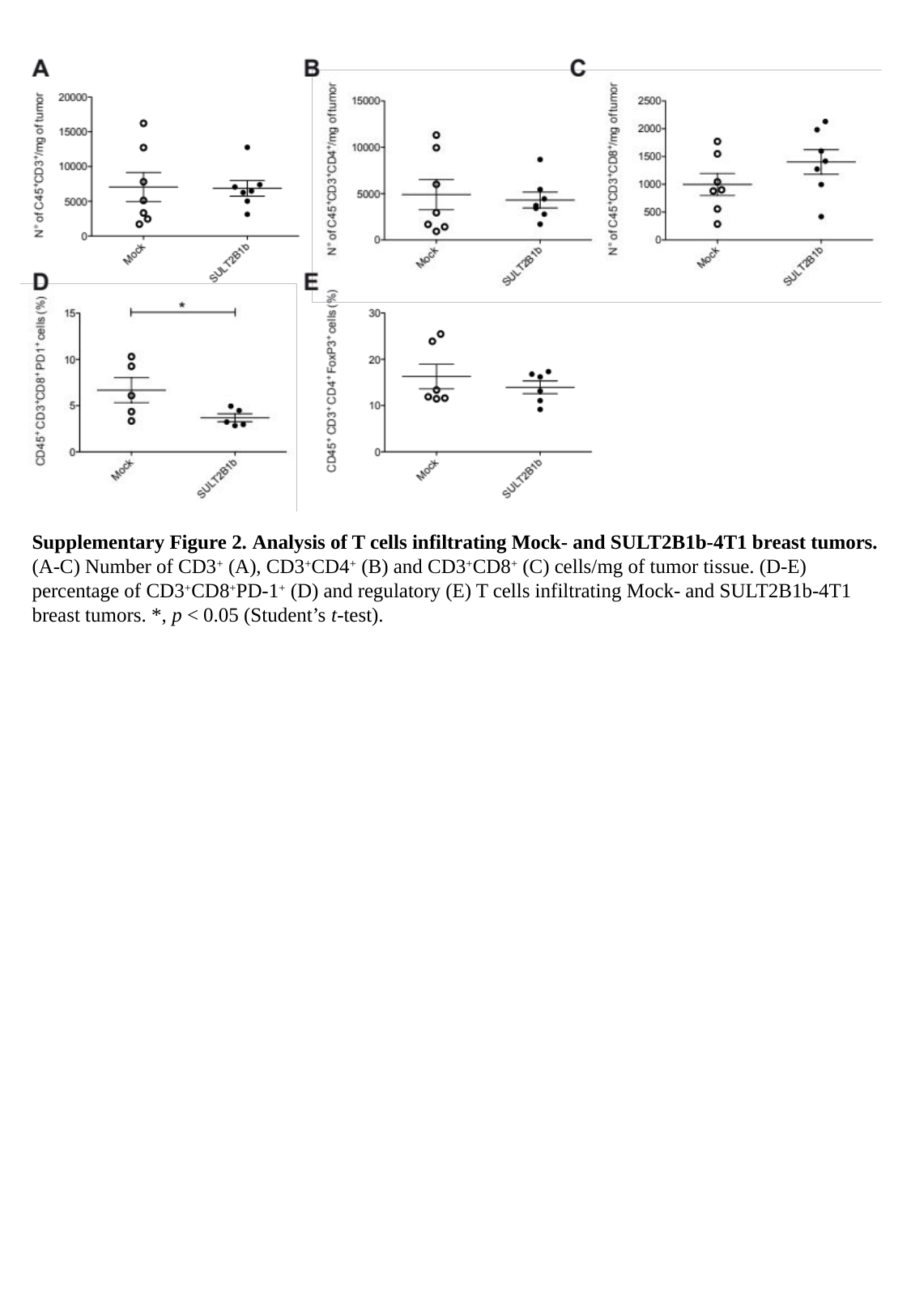

Supplementary Figure 2. Analysis of T cells infiltrating Mock- and SULT2B1b-4T1 breast tumors. (A-C) Number of CD3+ (A), CD3+CD4+ (B) and CD3+CD8+ (C) cells/mg of tumor tissue. (D-E) percentage of CD3+CD8+PD-1+ (D) and regulatory (E) T cells infiltrating Mock- and SULT2B1b-4T1 breast tumors. *, p < 0.05 (Student’s t-test).

## Slide 3
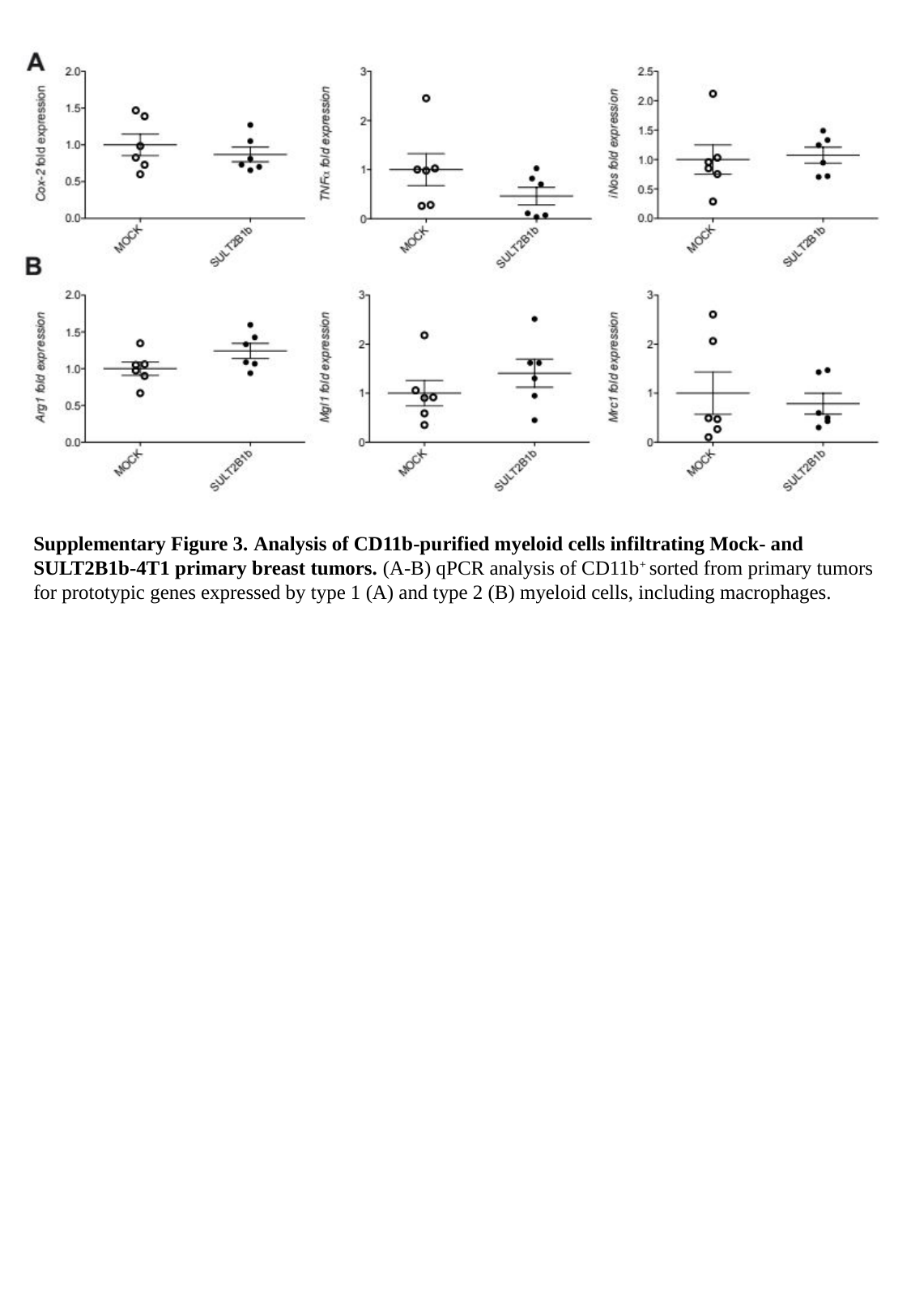

Supplementary Figure 3. Analysis of CD11b-purified myeloid cells infiltrating Mock- and SULT2B1b-4T1 primary breast tumors. (A-B) qPCR analysis of CD11b+ sorted from primary tumors for prototypic genes expressed by type 1 (A) and type 2 (B) myeloid cells, including macrophages.

## Slide 4
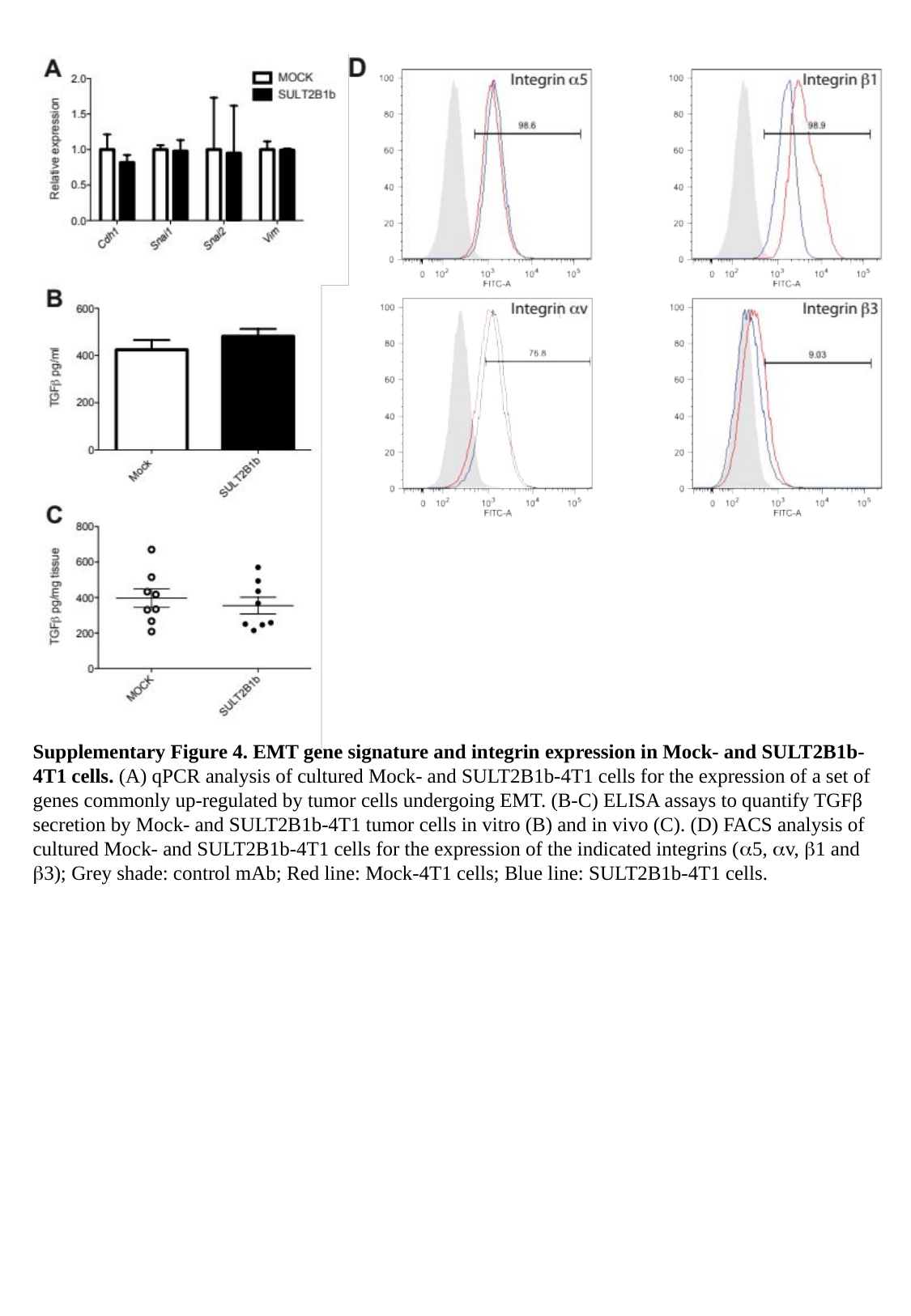

Supplementary Figure 4. EMT gene signature and integrin expression in Mock- and SULT2B1b-4T1 cells. (A) qPCR analysis of cultured Mock- and SULT2B1b-4T1 cells for the expression of a set of genes commonly up-regulated by tumor cells undergoing EMT. (B-C) ELISA assays to quantify TGFβ secretion by Mock- and SULT2B1b-4T1 tumor cells in vitro (B) and in vivo (C). (D) FACS analysis of cultured Mock- and SULT2B1b-4T1 cells for the expression of the indicated integrins (a5, av, b1 and b3); Grey shade: control mAb; Red line: Mock-4T1 cells; Blue line: SULT2B1b-4T1 cells.

## Slide 5
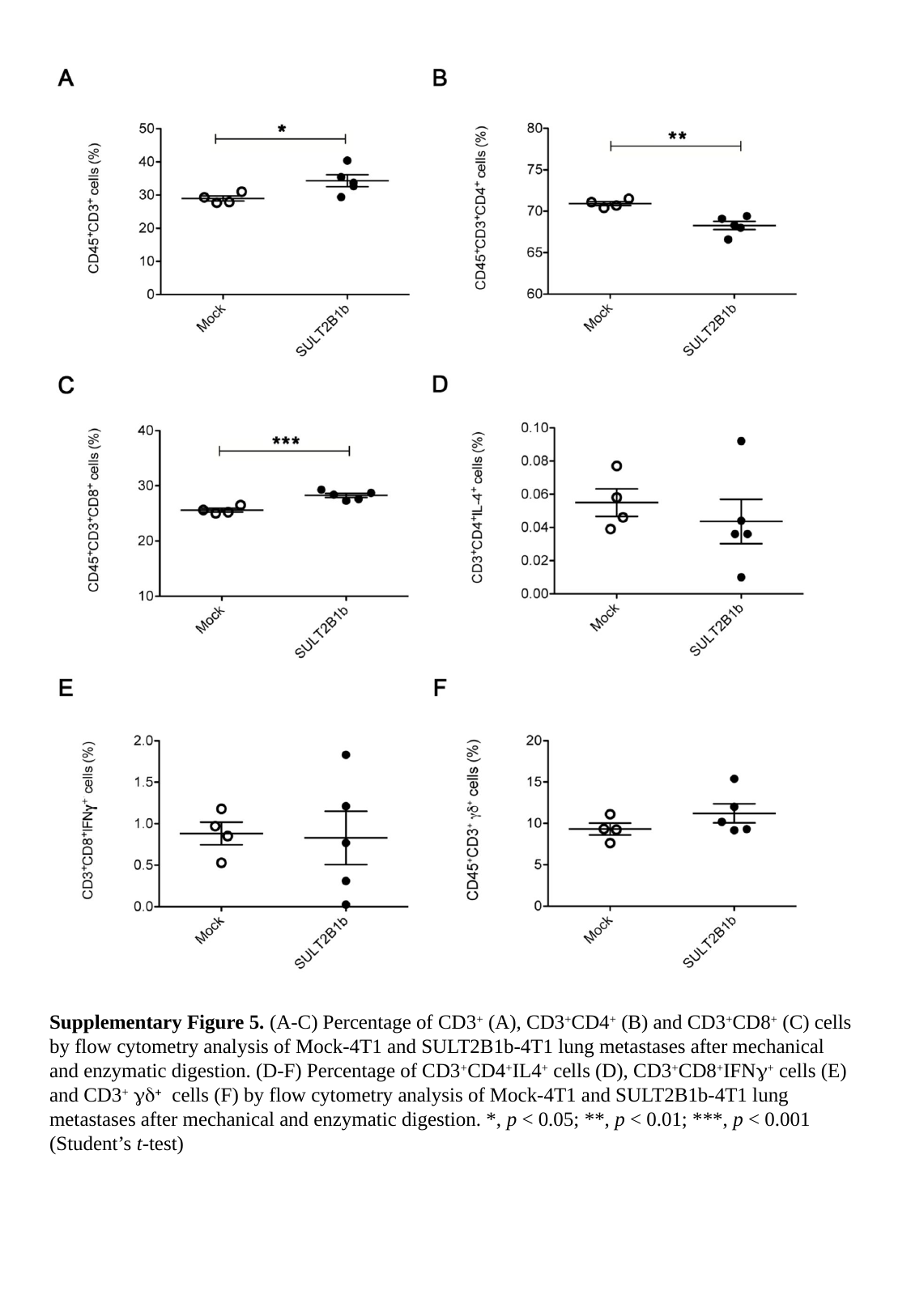

Supplementary Figure 5. (A-C) Percentage of CD3+ (A), CD3+CD4+ (B) and CD3+CD8+ (C) cells by flow cytometry analysis of Mock-4T1 and SULT2B1b-4T1 lung metastases after mechanical and enzymatic digestion. (D-F) Percentage of CD3+CD4+IL4+ cells (D), CD3+CD8+IFNγ+ cells (E) and CD3+ γd+ cells (F) by flow cytometry analysis of Mock-4T1 and SULT2B1b-4T1 lung metastases after mechanical and enzymatic digestion. *, p < 0.05; **, p < 0.01; ***, p < 0.001 (Student’s t-test)

## Slide 6
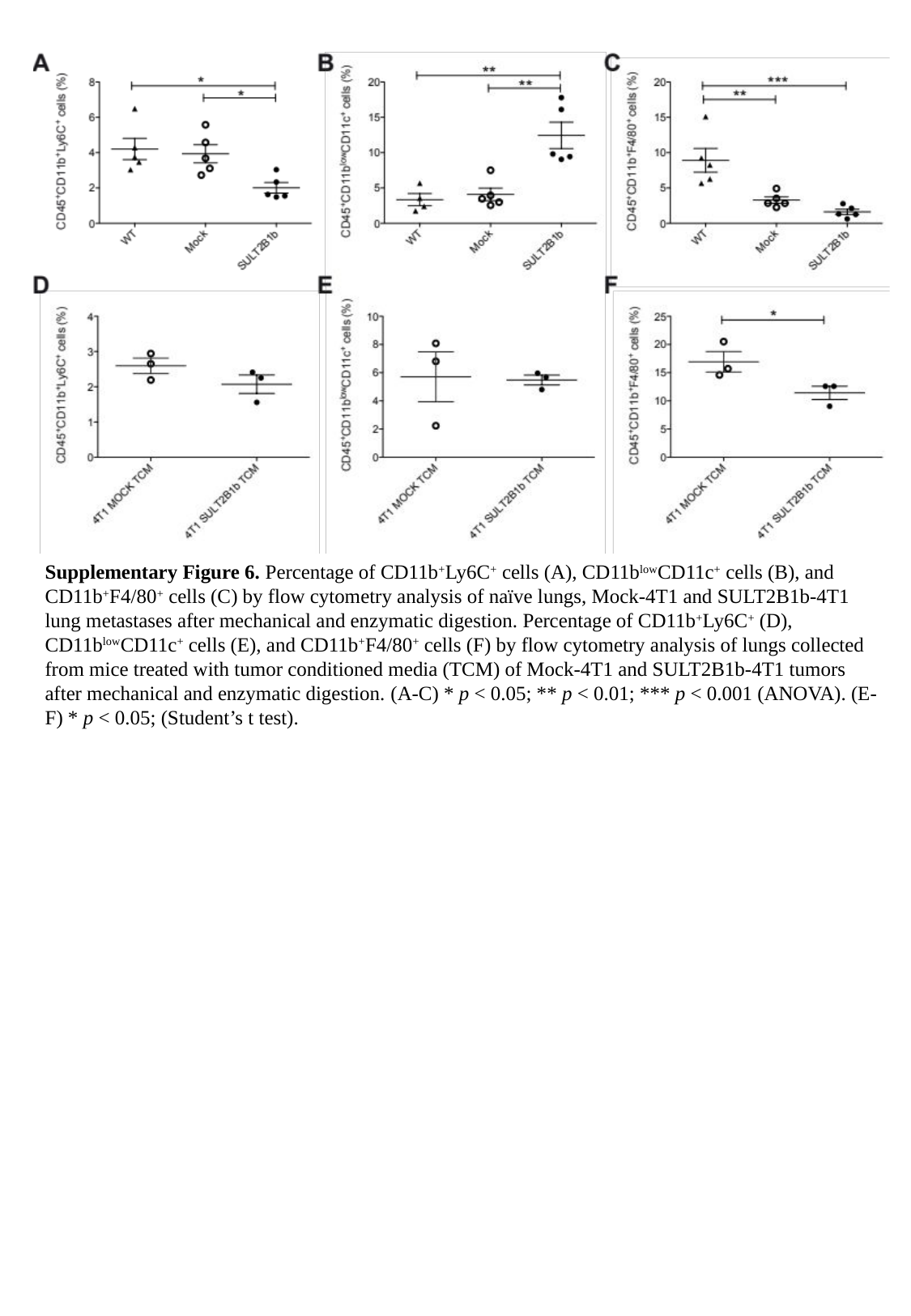

Supplementary Figure 6. Percentage of CD11b+Ly6C+ cells (A), CD11blowCD11c+ cells (B), and CD11b+F4/80+ cells (C) by flow cytometry analysis of naïve lungs, Mock-4T1 and SULT2B1b-4T1 lung metastases after mechanical and enzymatic digestion. Percentage of CD11b+Ly6C+ (D), CD11blowCD11c+ cells (E), and CD11b+F4/80+ cells (F) by flow cytometry analysis of lungs collected from mice treated with tumor conditioned media (TCM) of Mock-4T1 and SULT2B1b-4T1 tumors after mechanical and enzymatic digestion. (A-C) * p < 0.05; ** p < 0.01; *** p < 0.001 (ANOVA). (E-F) * p < 0.05; (Student’s t test).

## Slide 7
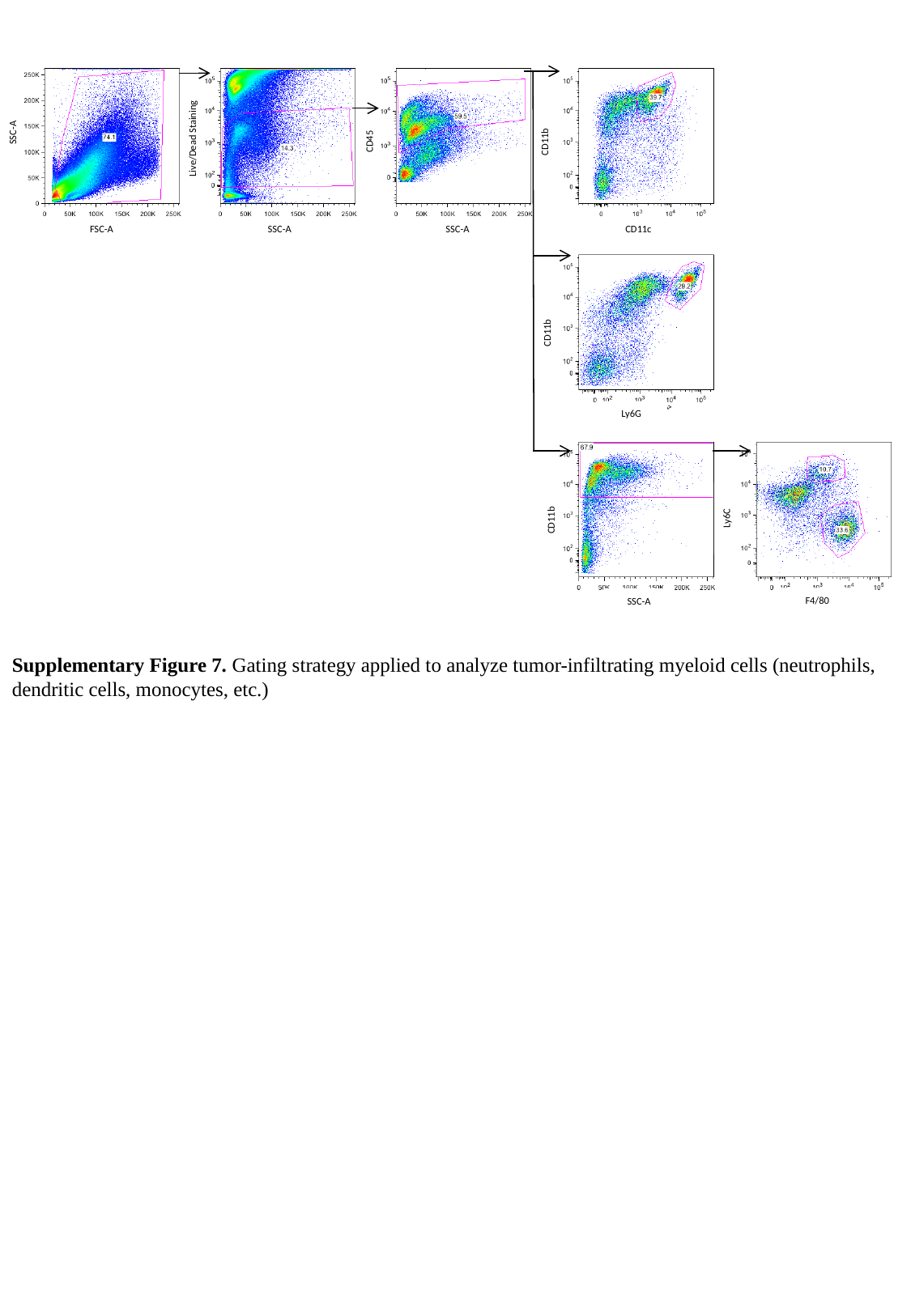

SSC-A
Live/Dead Staining
CD11b
CD45
FSC-A
SSC-A
SSC-A
CD11c
CD11b
Ly6G
Ly6C
CD11b
F4/80
SSC-A
Supplementary Figure 7. Gating strategy applied to analyze tumor-infiltrating myeloid cells (neutrophils, dendritic cells, monocytes, etc.)

## Slide 8
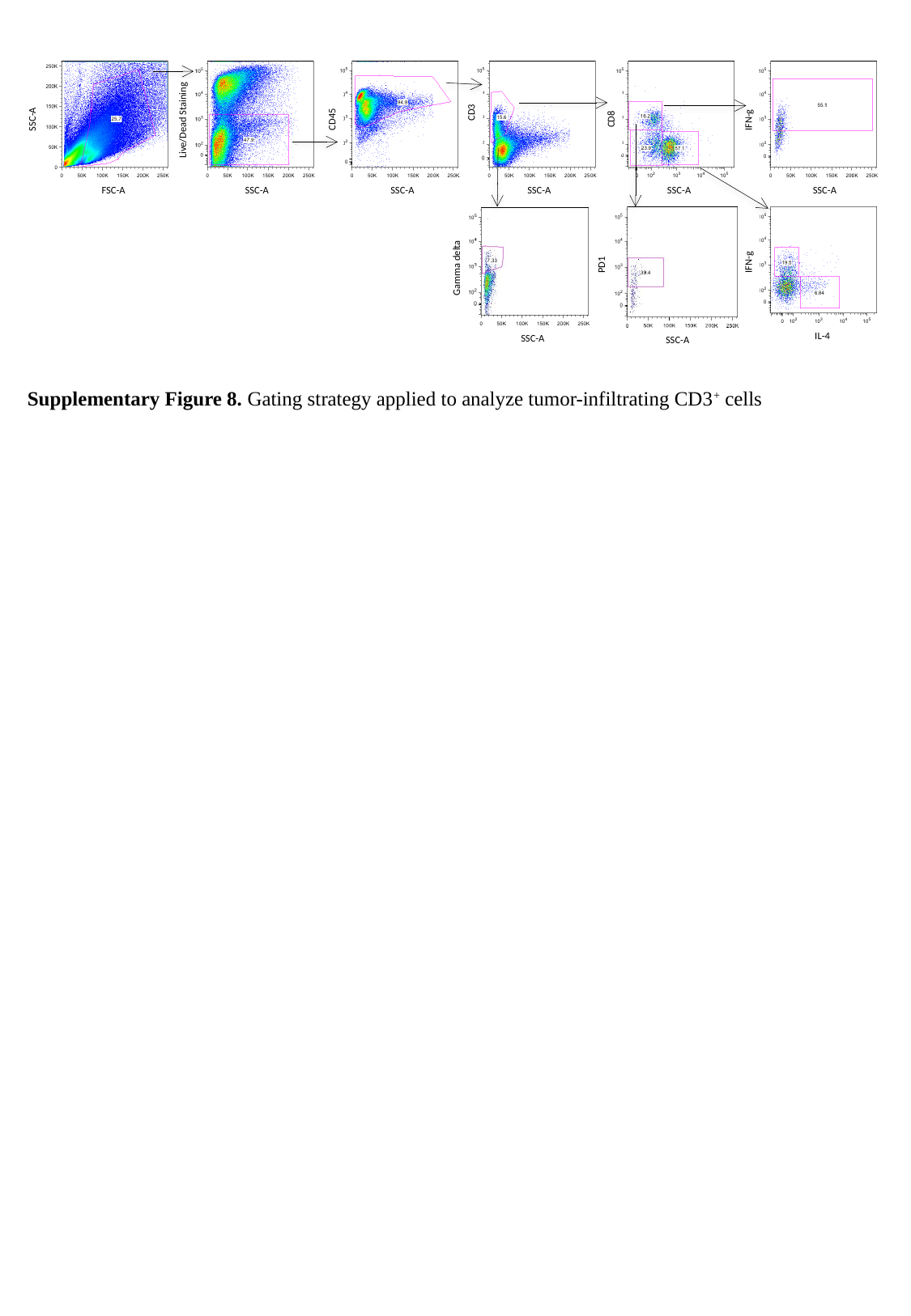

SSC-A
Live/Dead Staining
CD3
CD45
IFN-g
CD8
SSC-A
SSC-A
SSC-A
FSC-A
SSC-A
SSC-A
IFN-g
PD1
Gamma delta
IL-4
SSC-A
SSC-A
Supplementary Figure 8. Gating strategy applied to analyze tumor-infiltrating CD3+ cells

## Slide 9
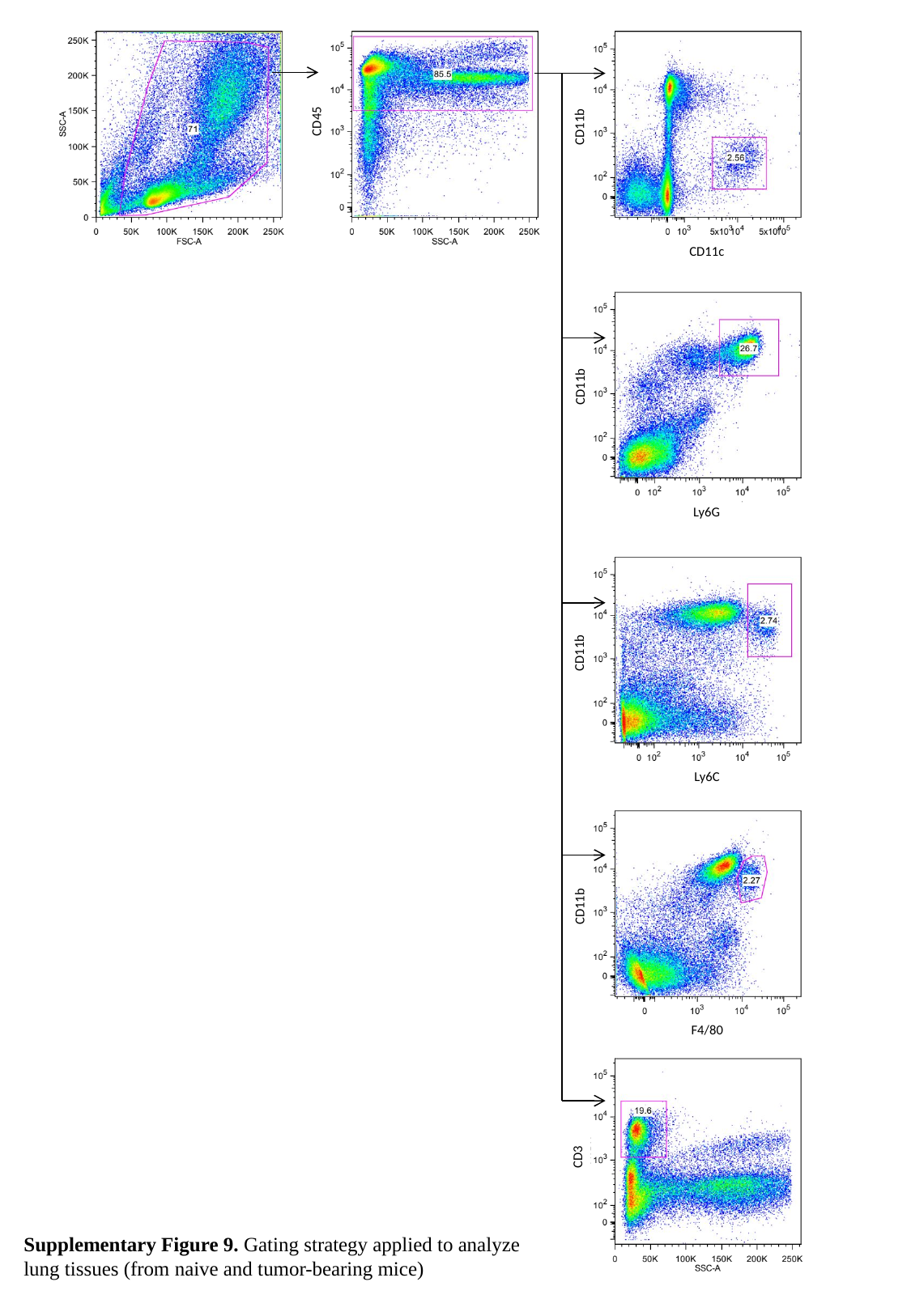

CD45
CD11b
CD11c
CD11b
Ly6G
CD11b
Ly6C
CD11b
F4/80
CD3
Supplementary Figure 9. Gating strategy applied to analyze lung tissues (from naive and tumor-bearing mice)
